# Mass drug administration of ivermectin in south-eastern Senegal reduces the survivorship of wild-caught, blood fed malaria vectors

**DOI:** 10.1186/1475-2875-9-365

**Published:** 2010-12-20

**Authors:** Massamba Sylla, Kevin C Kobylinski, Meg Gray, Phillip L Chapman, Moussa D Sarr, Jason L Rasgon, Brian D Foy

**Affiliations:** 1 Arthropod-borne and Infectious Diseases Laboratory, Department of Microbiology, Immunology & Pathology, Colorado State University, Fort Collins, Colorado, USA; 2 Department of Statistics, Colorado State University, Fort Collins, Colorado, USA; 3 Ministère de la Santé et de la Prévention Médicale, Dakar, Senegal; 4 The W. Harry Feinstone Department of Molecular Microbiology and Immunology, Bloomberg School of Public Health, Johns Hopkins University, Baltimore, Maryland 21205, USA; 5 The Johns Hopkins Malaria Research Institute, Bloomberg School of Public Health, Johns Hopkins University, Baltimore, Maryland, USA

## Abstract

**Background:**

In south-eastern Senegal, malaria and onchocerciasis are co-endemic. Onchocerciasis in this region has been controlled by once or twice yearly mass drug administration (MDA) with ivermectin (IVM) for over fifteen years. Since laboratory-raised *Anopheles gambiae *s.s. are susceptible to ivermectin at concentrations found in human blood post-ingestion of IVM, it is plausible that a similar effect could be quantified in the field, and that IVM might have benefits as a malaria control tool.

**Methods:**

In 2008 and 2009, wild-caught blood fed *An*. *gambiae *s.l. mosquitoes were collected from huts of three pairs of Senegalese villages before and after IVM MDAs. Mosquitoes were held in an insectary to assess their survival rate, subsequently identified to species, and their blood meals were identified. Differences in mosquito survival were statistically analysed using a Glimmix model. Lastly, changes in the daily probability of mosquito survivorship surrounding IVM MDAs were calculated, and these data were inserted into a previously developed, mosquito age-structured model of malaria transmission.

**Results:**

*Anopheles gambiae *s.s. (P < 0.0001) and *Anopheles arabiensis *(P = 0.0191) from the treated villages had significantly reduced survival compared to those from control villages. Furthermore, *An gambiae *s.s. caught 1-6 days after MDA in treated villages had significantly reduced survival compared to control village collections (P = 0.0003), as well as those caught pre-MDA (P < 0.0001) and >7 days post-MDA (P < 0.0001). The daily probability of mosquito survival dropped >10% for the six days following MDA. The mosquito age-structured model of malaria transmission demonstrated that a single IVM MDA would reduce malaria transmission (R_o_) below baseline for at least eleven days, and that repeated IVM MDAs would result in a sustained reduction in malaria R_o_.

**Conclusions:**

Ivermectin MDA significantly reduced the survivorship of *An. gambiae *s.s. for six days past the date of the MDA, which is sufficient to temporarily reduce malaria transmission. Repeated IVM MDAs could be a novel and integrative malaria control tool in areas with seasonal transmission, and which would have simultaneous impacts on neglected tropical diseases in the same villages.

## Background

Every year, an estimated 500 million people are afflicted with malaria worldwide, killing more than one million people, most of whom are children in sub-Saharan Africa [1,2]. Current control measures for preventing malaria transmission in Africa focus on the use of long-lasting insecticide-treated nets (LLITNs) treated with pyrethroids and indoor residual spraying (IRS) with organochlorines and pyrethroids. Both of these control methods have proven effective as a means for reducing *Plasmodium *transmission by endophagic malaria vectors but are potentially threatened by vector resistance to the currently used insecticides [3]. The development of novel methods to reduce *Plasmodium *transmission that can integrate with and enhance current malaria control measures, as well as other health priorities, is critical.

In 1987, ivermectin (IVM) was registered for human use for the control of onchocerciasis [4] and later for lymphatic filariasis [5]. Its primary effects are against microfilariae in the human body, which are the transmissible parasite stages of these diseases. Ivermectin has been used extensively since the mid-1990's in mass drug administration (MDA) campaigns across Africa by the African Programme for Onchocerciasis Control (APOC) [6] and the Global Program to Eliminate Lymphatic Filariasis (GPELF) [5]. Annually, more than 80 million people across the tropics are treated with IVM by MDA [7].

Previous *in vitro *and animal studies demonstrated that IVM can reduce the survivorship of multiple mosquito species after ingesting the drug in blood [8-11]. Wilson [12] reviewed these and other studies and suggested that the avermectins might impart their strongest impact on disease transmission by reducing vector longevity thereby reducing vectorial capacity. Concentrations of IVM found in human venous plasma after standard IVM MDA (150 μg/kg) reduced the survivorship and re-blood feeding frequency of laboratory-reared *Anopheles gambiae *s.s., which are the two most critical variables in models of vectorial capacity [13]. Two studies have directly blood fed laboratory-reared *Anopheles *spp. mosquitoes on humans who have ingested IVM. Foley *et al *[14] reported reduced *Anopheles farauti *survivorship when mosquitoes fed on one person who ingested 250 μg/kg of IVM. Chaccour *et al *[15] found that *An. gambiae *s.s. blood fed on humans one day after they had ingested 200 μg/kg of IVM had significantly reduced survivorship, but the effect was not apparent fourteen days post-ingestion. To date, the only field based study on the effects of IVM against wild mosquitoes was performed in Papua New Guinea and focused on lymphatic filariasis control. Bockarie *et al *[16] demonstrated that MDA with IVM (400 μg/kg) in combination with diethylcarbamazine citrate (6 mg/kg) in one village reduced the survivorship of wild *Anopheles punctulatus *up to four days after MDA. In another village, MDA with IVM (400 μg/kg) alone reduced the survivorship of wild *An. punctulatus *captured the day after MDA [16].

The goal of this study was to determine if IVM MDA of humans in Senegal for onchocerciasis control could simultaneously reduce the survivorship of wild African malaria vectors, and if so, for how long this effect would occur, and to model this effect on malaria transmission. Villages in south-eastern Senegal have been treated once or twice yearly with IVM MDA (150 μg/kg) for onchocerciasis eradication for over fifteen years [17]. This same region has hyperendemic malaria transmission [18,19] and has an abundant and diverse *Anopheles *malaria vector fauna [20]. In 2008 and 2009, blood-fed *Anopheles *spp. were captured from inside peoples' huts before and after IVM MDA in three replicate pairs of villages in south-eastern Senegal. Survivorship of the mosquitoes was assessed by holding them in a field insectary for five days. Mosquito survivorship data were then incorporated into a modified previously-developed model [21] to evaluate the potential of IVM to reduce malaria transmission. The results demonstrate that IVM MDA reduces the survivorship of wild *An. gambiae *s.s. and that this reduction in survivorship should be sufficient to reduce malaria transmission.

## Methods

### Study site

The study was conducted in the villages of Boundacoundi, Damboucoye, Nathia, Ibel and Ndebou, all in the Sudano-Guinean zone of rural south-eastern Senegal. The five villages are located along a 15 km stretch of road heading west out of Kedougou. Most of the people in this area are subsistence farmers. They live in extended family compounds with 2-10 sleeping huts, and cultivate maize, sorghum and groundnuts between these compounds and in separate fields outside the village. Cattle, sheep, goats, dogs, and chickens are the primary domesticated animals in the villages. In 2008, two villages were sampled, Ibel and Ndebou. Ibel was treated by MDA with 150 μg/kg of IVM (Mectizan™, Merck & Co., Inc) on August 8, 2008, while Ndebou was not treated and served as the paired control village. In 2009, two pairs of villages were sampled. Ndebou and Boundacoundi were the first pair of villages sampled and MDA occurred on August 6, 2009 in Ndebou, with Boundacoundi serving as the control. Damboucoye and Nathia were the second pair of villages sampled and MDA occurred on October 11, 2009 in Damboucoye, with Nathia serving as the control. MDA was coordinated by APOC in Senegal and the Senegalese Ministry of Health, and performed through community-directed treatment by the local nurses. Permission to conduct mosquito sampling surrounding these MDAs was granted first by the Senegalese Ministry of Health and then by the residents of each village. The study was also reviewed by the Colorado State University Institutional Review Board prior to being conducted.

### Mosquito collections

Indoor resting, wild, blood fed *Anopheles *mosquitoes were collected in the morning from huts people had slept in the previous night using backpack aspirators (John W. Hock, Gainesville, FL, USA). After capture, *Anopheles *mosquitoes were transferred by mouth aspirators from backpack aspirator cups to 473 ml cardboard containers screened with organdy. The containers were labelled and designated by village, date collected, and the specific hut from which mosquitoes were collected. Containers were placed into a large basket and two moist towels were placed over the top of the basket to keep the mosquitoes humid and cool. Immediately following morning aspirations, the mosquitoes were transported back to the insectary in Bandafassi (2008) or Kedougou (2009) and maintained on shelves. Insectaries were designated rooms of houses and had screened and slatted windows so that they naturally fluctuated with the ambient temperature and humidity. Temperature and humidity within the insectary ranged from (27 - 30°C) and (66 - 86%). Any dead, non-blood fed, or non-*Anopheles *mosquitoes were removed from the containers upon placement in the insectary. The containers had a moistened sponge and raisins placed on top to serve as water and sugar sources for the mosquitoes. Survivorship was checked daily at 12:00 pm and dead mosquitoes were removed from the containers. The containers were then randomly placed back onto the shelves. All mosquitoes that survived for five days were frozen and counted as alive on day five post-capture. In 2009, both the treated and the control village were sampled on the same day by two separate field teams. In 2008, only one village was sampled on each day (Additional file 1).

### Mosquito processing

Mosquitoes were identified morphologically to species [22,23] in the field insectary immediately following their death. The abdomens were separated from the thoraxes of all *Anopheles *spp. and placed into two separate 1.5 ml microfuge tubes containing the silica gel desiccant T.H.E. (EMD Chemicals, NJ, USA). The tubes were labelled with the village, date of aspiration and hut location, and all relevant information was recorded on matching log sheets. Processed mosquitoes were shipped back to Colorado State University for further molecular analysis. DNA was extracted with the Qiagen DNeasy kit (Qiagen Sciences, Maryland, USA) and a Qiacube robot (Qiagen Sciences, Maryland, USA). Multiplex polymerase chain reaction was used to molecularly identify members of the *An. gambiae *s.l. complex [24]. A subset of twelve or fewer *An. gambiae *s.l. from each collection day that died within one day of capture and contained undigested blood at the time of processing had their blood meals analysed by the multiplex polymerase chain reaction to determine the source of blood [25].

### Survivorship model and statistics

**Figure 1:**
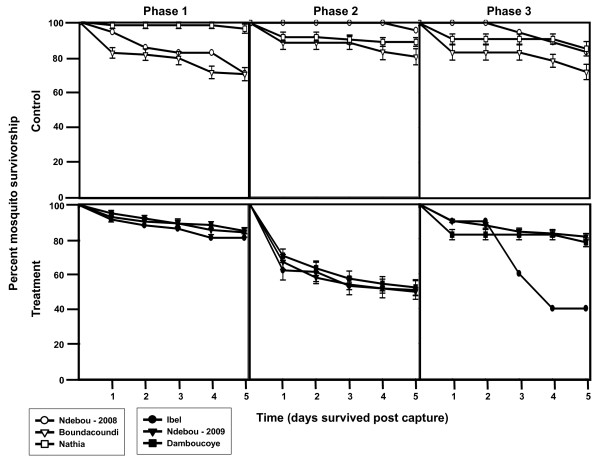
**Percent survivorship of aspirated *An. gambiae *s.s. grouped by treatment and phase**. Percent survivorship of all aspirated *An. gambiae *s.s. (N = 1265) held five days post-capture from all three replicates. Standard error bars represent the percent survivorship variation of all mosquitoes from each village grouped within each treatment and phase.

A generalized linear mixed (Glimmix) model was used to assess the effects of IVM MDA on mosquito survivorship. Mosquito survivorship results for each village at each sampling date were classified by treatment (whether or not they originated from a treatment or control village), replicate (the three pairs of villages sampled over the two field seasons), and phase. The three phases are groups of sampling dates from each village before MDA (phase 1), one to six days after MDA (phase 2), and seven days or more after MDA (phase 3). The one to six day grouping immediately after MDA was chosen based on the differential survivorship curves generated from the three replicates (Figure 1). Treatment and phase were treated as fixed effects. Replicates were treated as random effects with sample date nested within replicate and phase. The model was fit by the SAS Glimmix procedure using maximum likelihood estimation with three Gaussian quadrature points (SAS Institute, Cary, NC, USA). The percent survivorship of mosquitoes was tested for interaction of treatment by phase, and if significant, then post-tests were performed to determine which treatment by phase groups significantly differed from the others. The efficacy of IVM to reduce mosquito survivorship would be reflected by a significant drop in the treated village survivorship at the phase 2 group compared to control and pre-treatment groups.

### Modelling the change in the basic reproductive number of malaria due to ivermectin mass drug administrations

A linear calculation of the daily probability of survivorship was determined from the five-day survivorship values of *An. gambiae *s.l. captured before and after MDA from all three replicates. These daily probability of survivorship estimates were then inserted into an age-structured model of mosquito population dynamics from Billingsley *et al *[21] with several minor modifications. The proportion of treated humans was not held constant, but rather varied temporally based on MDA coverage. It was assumed that once treated with drug, humans remained mosquitocidal to feeding *Anopheles *for six days. Output of this model was used as input for an age-structured model [21] showing the relative change in the basic reproductive number of malaria during IVM treatment. A relative R_0 _< 1 indicates a decrease in transmission, while relative R_0 _> 1 indicates an increase in transmission and relative R_0 _= 1 indicates no change.

Immature mosquito lifestages were assumed to have a daily survival rate of 0.9. Based on the data estimated from field-collected mosquitoes, it was assumed that mosquitoes that imbibed blood from an untreated human had a daily survival rate of 0.96, while mosquitoes that fed on a treated human had a daily survival rate of 0.86 for 3 days post-feeding. To be conservative, it was assumed that after feeding on a treated human, mosquitoes experienced a decrease in their daily survival rate for three days post-feeding, after which they recovered to untreated levels. An extrinsic incubation period of fourteen days was used for the model. Age-specific fecundity was not affected by IVM as was previously stated [21].

## Results

### Mosquito survivorship analysis

**Figure 2:**
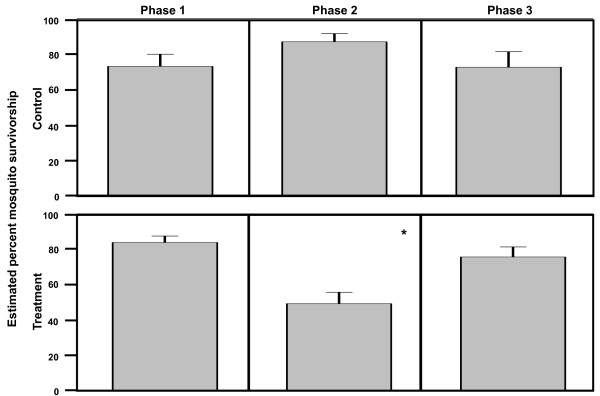
**Glimmix model estimated percent survivorship of *An. gambiae *s.s. grouped by treatment and phase**. Glimmix model estimated percent survivorship of all *An. gambiae *s.s. (N = 1265) held five days post-capture from all three replicates. * Treatment by phase was significant (F-value = 18.27, P < 0.0001). Treatment at phase 2 significantly differed from control at phase 2 (t-value = 4.01, P = 0.0003), treatment at phase 1 (t-value = 8.31, P < 0.0001), and treatment at phase 3 (t-value = -4.61, P < 0.0001).

Figure 1 depicts the percent survivorship of *An. gambiae *s.s. from all three replicates grouped by treatment and phase. There is an observable reduction in *An. gambiae *s.s. survivorship after IVM MDA (phase 2) in the treated villages that lasts for six days. The survivorship of *An. gambiae *s.s. in Ibel during phase 3 is low (Figure 1), but this line only represents ten mosquitoes caught from one collection (Additional file 1). A total of 1,265 *An. gambiae *s.s. from three replicates were captured and held for survivorship analysis. The model of estimated mosquito survivorship for *An. gambiae *s.s. identified a treatment by phase interaction, indicating that the difference between treated and control survivorship depends on phase (F-value = 18.27, P < 0.0001) (Figure 2). In follow-up comparisons, treatment at phase 2 significantly differed from control at phase 2 (t-value = 4.01, P = 0.0003), and it also significantly differed from both treatment at phase 1, pre-MDA (t-value = 8.31, P < 0.0001) and treatment at phase 3, seven days and after IVM MDA (t-value = -4.61, P < 0.0001). The conclusion of this analysis is that IVM MDA significantly reduced the survivorship of *An. gambiae *s.s. for six days past the date of the MDA.

**Figure 3:**
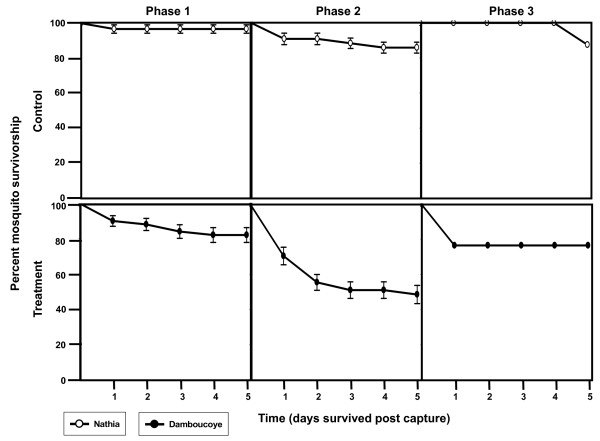
**Percent survivorship of aspirated *An. arabiensis *grouped by treatment and phase**. Percent survivorship of all aspirated *An. arabiensis *(N = 153) held five days post-capture from Nathia and Damboucoye. Standard error bars represent the percent survivorship variation of all mosquitoes from each village grouped within each treatment and phase.

**Figure 4:**
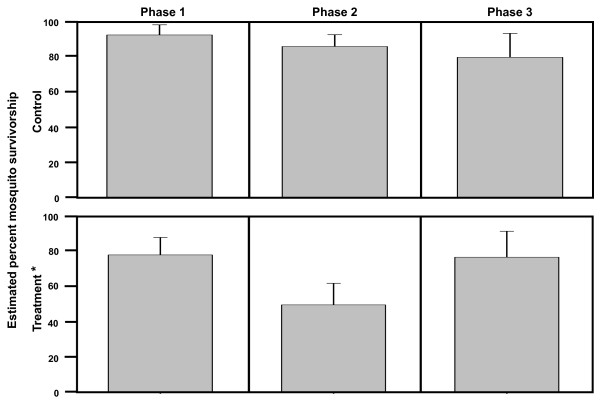
**Glimmix model estimated percent survivorship of *An. arabiensis *grouped by treatment and phase**. Glimmix model estimated percent survivorship of all *An. arabiensis *(N = 153) held five days post-capture from Nathia and Damboucoye. *Treatment was significant (F-value = 7.01, P = 0.0191).

Adequate numbers for survivorship analysis of *An. arabiensis *were only caught during the third replicate (Damboucoye and Nathia, n = 153). There appears to be a reduction in survivorship of *An. arabiensis *following IVM MDA (Figure 3), but treatment by phase comparisons were not significantly different from each other (F-value = 0.66, P = 0.5332). However, treatment alone was significant (F-value = 7.01, P = 0.0191), therefore the overall survivorship of *An. arabiensis *was lower in the treated village compared to the control village (Figure 4).

### Blood meal analysis

**Figure 5:**
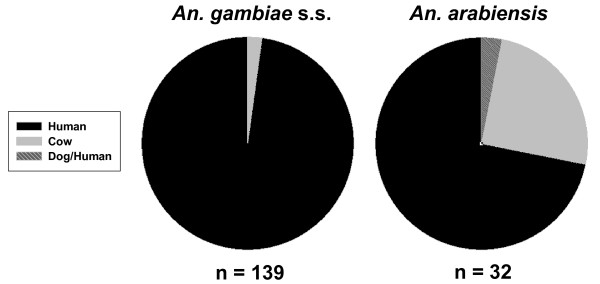
**Percent composition of blood meal sources of aspirated *An. gambiae *s.l**. The percent blood meal sources of a subset of *An. gambiae *s.s. (n = 139) and *An. arabiensis *(n = 32) aspirated from all five villages in 2008 and 2009.

Almost all, 97.8% (136/139), of *An. gambiae *s.s. blood meals that were analysed were from humans. Based on this information it was assumed that almost all of the *An. gambiae *s.s. held for the study had fed on humans. However, only 75% (24/32) of analysed *An. arabiensis *blood meals were from humans (Figure 5).

### Modelling the effect of ivermectin treatment on malaria transmission

**Figure 6:**
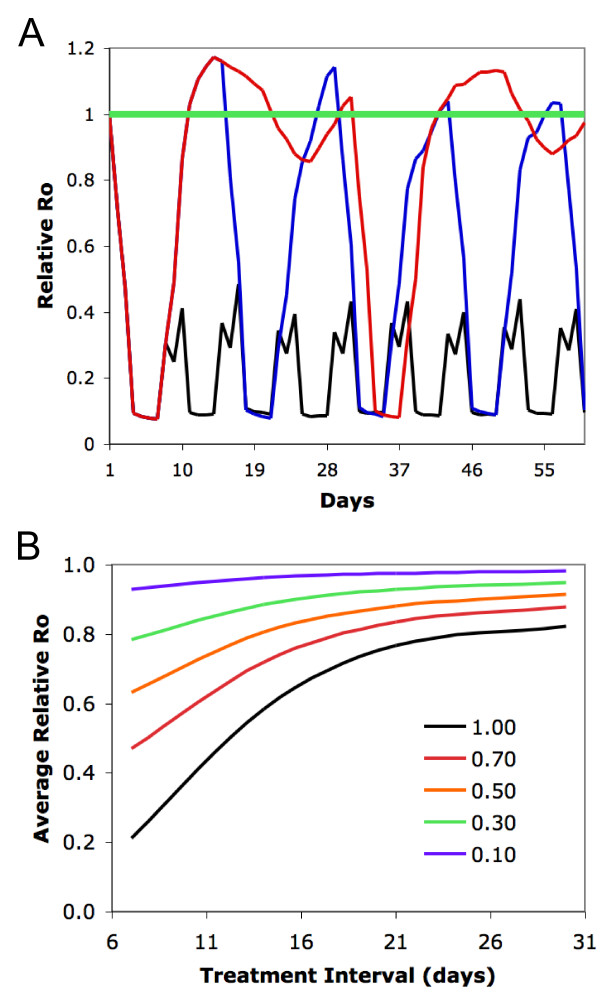
**Predicted relationship between MDA and changes in malaria R_0_**. Relative malaria R_0 _values < 1.0 indicate reduction in transmission potential, values > 1.0 indicate increase in transmission potential, while 1.0 (green line in 6A) indicates no change. A) Ideal situation of 100% coverage with MDA, administered every 7 (black), 14 (blue) or 30 (red) days. B) Average relative R_0 _for a range of MDA coverage levels (10% to 100%) and treatment regimes (every 7 to 30 days).

Simulations indicate that MDA with IVM can significantly reduce R_0 _for a short period of time after drug administration (Figure 6A). Using the previously stated assumptions and an ideal situation of 100% coverage, a synchronized MDA of IVM results in an approximately 90% reduction in R_0 _immediately following MDA (Figure 6A). After effective drug concentrations are cleared from the blood of the human population, R_0 _rebounds to pre-treatment levels or slightly higher if no further MDAs are performed. To keep relative R_0 _levels at significantly lower levels, drug treatment must be administered repeatedly. Less frequent treatments allow for periods of control alternated with periods of no control (Figure 6A).

Due to the fluctuating nature of the control dynamics, where R_0 _is changing on a daily basis, it is more informative to compare average relative R_0 _between different treatment intervals. This can be easily calculated by summing the area under the curve for both pre and post-treatment scenarios. Under the ideal conditions of 100% coverage with treatment every week, mean R_0 _can be reduced by 80%. Lower levels of coverage or less frequent MDAs reduce the efficacy of this strategy, but in all cases MDA resulted in some level of control (Figure 6B).

## Discussion

Previously published laboratory based evidence showed that colonized *An. gambiae *s.s. is susceptible to IVM at concentrations relevant to human pharmacokinetics after a typical MDA [13], and that colonized *An. gambiae *s.s. fed on IVM-treated humans one day post-treatment had reduced survivorship [15]. The current study now demonstrates that routine MDA of IVM to people significantly reduces the survivorship of wild *An. gambiae *s.s. for up to six days post MDA. This six day lethal effect is longer than the two days observed from Kobylinski *et al*. [13], and this effect occurs despite incomplete MDA coverage in treated villages. Three field replicates were performed over space and time in different villages to make this study a rigorous assessment of the effects of IVM MDA on *An. gambiae *s.l.

There were no significant differences in *An. arabiensis *treatment by phase survivorship (F-value = 0.66, P = 0.5332) but this was almost certainly due to sampling, in that adequate numbers (n = 153) for survivorship analysis were only captured in the third replicate MDA. The third replicate MDA was performed in October 2009, toward the end of the rainy season when *An. arabiensis *is more prevalent [20]. There was a 38% reduction in mosquito survivorship from phase 2 treatment collections compared to phase 2 control collections (Figure 4). The fact that treatment alone was significant (F-value = 7.01, P = 0.0191), means that the overall survivorship of *An. arabiensis *was lower in the treated village compared to the control village (Figure 4). Furthermore, only 75% (24/32) of *An. arabiensis *blood meals were from humans (Figure 5), which reduces the probability that mosquitoes held for survivorship analysis may have ingested an IVM-containing blood meal. Fritz *et al *[26] reported that colonized *An. gambiae *s.s. and colonized *An. arabiensis *have almost identical susceptibility to IVM. When these data are considered together, it is reasonable to assume that upon further replication wild *An. arabiensis *will be shown to be as susceptible to IVM MDA as wild *An. gambiae *s.s.

Based on clinical records, 84.2% (203/241) of people in Damboucoye and 82.1% (311/379) of people in Ndebou were treated with IVM during these two MDAs. Pregnant women and children under 90 cm did not receive the drug, following the manufacturer guidelines. Mosquitoes that were held for survivorship analysis for five days had completely digested their blood meals, which made it impossible to detect IVM from individual mosquitoes. Yet it is impressive that mosquito survival was still significantly reduced despite not knowing whether any one mosquito fed on a treated person. Incomplete coverage may actually be beneficial to the overall concept of repeated IVM MDAs for malaria control in that it may provide a refugia of untreated human hosts for mosquitoes to feed on which could reduce the likelihood of IVM resistance development in the mosquito population.

Of people accounted, 78.2% (903/1,155) utilized ITNs across the four villages surveyed in 2009. Even with high ITN coverage, human blood fed *An. gambiae *s.s. and *An. arabiensis *were frequently collected from the inside of huts, demonstrating that ITNs have limitations in preventing *Anopheles *from feeding on people in huts. Exophagic and exophilic malaria vectors also comprise an important part of the malaria transmission cycle in this study area; *Anopheles funestus *group mosquitoes are almost twice as likely to blood feed outdoors than indoors [20], and *Anopheles nili *tend to be exophilic or immediately exit huts after biting [20,27]. ITNs may reduce malaria transmission by exophagic vectors [28-30], but their primary efficacy is against endophagic vectors. It has also been shown that ITNs may shift vector host seeking times to earlier in the evening when people will not be sleeping under an ITN [31]. IRS will only affect the survivorship of endophilic vectors that contact the sprayed surfaces, and it is believed that the exophilic portion of the *An. gambiae *s.l. population led to the failure of IRS to eliminate malaria transmission during the Garki project [32]. Furthermore, a number of malaria vectors will naturally feed at dusk and dawn, when humans are less likely to be indoors and protected by an ITN or by IRS. Ivermectin MDA may be one of the few methods that can directly target these exophagic, exophilic, and crepuscular-feeding malaria vectors, and should integrate well with the employment of existing in-home control methods like ITNs and IRS.

Ivermectin has a different mode of action from the insecticide classes currently used for ITNs and IRS (i.e., carbamates, pyrethroids, and organochlorines) [3], in that it agonizes the glutamate-gated chloride anion channels found in invertebrate postsynaptic neurons and neuromuscular junctions [33,34]. This action hyperpolarizes the neurons and muscle fibers, leading to flaccid paralysis and insect death [35-37]. Once or twice yearly IVM MDA has been occurring in this region for over fifteen years [17], so the fact that a reduction in survivorship of *An. gambiae *s.s. was still detectable is a promising sign that resistance by *Anopheles *spp. may be slow to develop against this drug. Furthermore, the novel mode of action of ivermectin compared the currently used insecticides for malaria control should potentially minimize issues of cross-resistance where IVM MDA may be used in combination with IRS and ITNs.

*Anopheles gambiae *s.s. often requires two blood meals to complete its initial gonotrophic cycle [38,39] and thereafter will often take multiple blood meals per gonotrophic cycle [40-42] and feeds almost exclusively on humans (Figure 5) [20]. These blood feeding characteristics, coupled to the fact that the extrinsic incubation period for *Plasmodium *spp. is 9-14 days, means that most malaria transmission by *An. gambiae *s.s. will occur only after *Plasmodium *parasite-harbouring mosquitoes have taken multiple non-sporozoite transmitting blood meals from humans [21,43]. If human population clusters were simultaneously treated with IVM MDA, then most adult *An. gambiae *s.s. in the MDA area would imbibe a concentration of IVM that would reduce their survivorship. The predicted effect has the potential to temporarily shift the *An. gambiae *s.s. population age structure, which would reduce the reservoir of adult sporozoite-transmitting *An. gambiae *s.s. in the MDA area. The low sporozoite rate in the resulting mosquito population would temporarily reduce the basic reproductive number (R_0_) of malaria below the base number for approximately eleven days post IVM MDA. Current IVM MDAs for onchocerciasis control in Africa are performed only once or twice per year, and do not always coincide with local malaria transmission seasons. Such current applications would not be expected to lower malaria transmission long enough to see any noticeable reductions of parasite prevalence, intensity or disease in people. Indeed, malaria is hyperendemic in APOC-control areas of south-eastern Senegal despite IVM MDAs for more than 15 years. However, if IVM MDA is administered repeatedly, R_0 _can be reduced for an extended period of time.

This model, like all models, makes assumptions that may not be realistic in nature, such as homogeneous mosquito biting, no spatial structure, and the lack of density-dependent effects. However, the model results are conservative, since it only incorporates the direct IVM-related mortality effect observed in the field. Kobylinski *et al *[13] demonstrated in the lab that multiple sub-lethal IVM containing blood meals compounds mosquito mortality, but the model assumes complete recovery of surviving mosquitoes three days after ingesting an IVM-containing blood meal. Kobylinski *et al *[13] also demonstrated that the re-blood feeding frequency and blood digestion of *An. gambiae *s.s. are delayed after imbibing relevant IVM concentrations. Finally, Fritz *et al *[26] and personal observations have witnessed mosquito knockdown effects immediately after drug ingestion. All of these negative sub-lethal effects would probably lead to a further reduction in *An. gambiae *s.s. survivorship in the field, due to desiccation, predation, or insufficient nutrition, beyond that of outright mortality induced by IVM. Thus, it may be that the age-structured model is an underestimate of the true effect of IVM MDA on malaria transmission.

The age-structured model predicts that strategically administered, repeated IVM MDAs would achieve sustained reductions in malaria transmission. Such repeated MDAs may only be logistically feasible for areas with seasonal malaria transmission, such as the Sahel, or in areas experiencing malaria epidemics. An expanded IVM MDA regimen fits well with the idea of combating polyparasitism in some of these same communities [44,45]. Malaria and soil-transmitted helminths (STHs - *Ascaris lumbricoides, Trichuris trichiura, Strongyloides stercoralis*, and hookworms) are co-endemic across much of sub-Saharan Africa [46-50]. The annual/biannual IVM MDAs for onchocerciasis control are not generally sufficient for controlling STHs because of relatively rapid re-infections due to their transmission dynamics [51-55]. Repeated IVM MDAs are likely to result in reductions of the prevalence and intensities of STHs in individuals receiving the drug [52-59], and would increase the personal incentive to participate in such MDAs. The combination of malaria and soil-transmitted helminth infections can exacerbate anaemia, resulting in worsened child development and more adverse pregnancy outcomes than these diseases cause on their own [60-64]. Therefore, the combined effects of IVM MDA on multiple parasites could potentially reduce anaemia in the human population which would lead to an overall improvement in human health beyond what would be expected from malaria control alone.

Future field work will need to be conducted to determine if repeated IVM MDAs can quantifiably reduce malaria transmission and if the model created here is an over or underestimate of what would occur in the field. Important to this future work is determining whether a logistically feasible IVM MDA interval can be devised that would reduce malaria transmission below a critical threshold and not foster IVM resistance in either *Anopheles *vectors or human nematode parasites.

## Conclusions

This study demonstrates that IVM MDA reduces the survivorship of wild-caught *An. gambiae *s.s. for up to six days post MDA and the modelled field data shows that repeated IVM MDAs should result in a sustained reduction of malaria transmission. Ivermectin MDA would be both a novel insecticide class and delivery method for reducing vector survivorship. It should also affect exophagic, exophilic, and crepuscular-feeding malaria vectors not normally targeted by malaria control measures. Repeated IVM MDAs should concomitantly reduce STH prevalence and intensities, which would further benefit human health beyond that of malaria control alone.

## Competing interests

The authors declare that they have no competing interests.

## Authors' contributions

MS, KCK, and BDF designed research; MS, KCK, MG and BDF performed research; BDF contributed reagents; MS, KCK, MDS and BDF coordinated field studies, MS, KCK, PLC, JLR and BDF analysed data/developed models; MS, KCK, JLR and BDF wrote the paper; MS, KCK, MG, PLC, JLR and BDF edited the paper. All authors read and approved the final manuscript.

## Supplementary Material

Additional file 1 **Number of mosquitoes captured by aspiration by mosquito species, village, year, phase, and date**. The number of mosquitoes caught each aspiration collection by date and grouped by phase. *Coll - number of aspiration collections performed per phase. **Trtmt - whether or not a village was treated by IVM MDA. ***#ct - number of each mosquito species caught that aspiration collection. Click here for file
